# Artificial Transcription Factors for Tuneable Gene Expression in *Pichia pastoris*

**DOI:** 10.3389/fbioe.2021.676900

**Published:** 2021-08-09

**Authors:** Gita Naseri, Kevin Prause, Housam Haj Hamdo, Christoph Arenz

**Affiliations:** ^1^Institute of Biology, Humboldt Universität zu Berlin, Berlin, Germany; ^2^Institute of Chemistry, Humboldt Universität zu Berlin, Berlin, Germany

**Keywords:** artificial transcription factor, metabolic engineering, *Pichia pastoris*, *Saccharomyces cerevisiae*, synthetic biology

## Abstract

The non-conventional yeast *Pichia pastoris* (syn. *Komagataella phaffii*) has become a powerful eukaryotic expression platform for biopharmaceutical and biotechnological applications on both laboratory and industrial scales. Despite the fundamental role that artificial transcription factors (ATFs) play in the orthogonal control of gene expression in synthetic biology, a limited number of ATFs are available for *P. pastoris*. To establish orthogonal regulators for use in *P. pastoris*, we characterized ATFs derived from Arabidopsis TFs. The plant-derived ATFs contain the binding domain of TFs from the plant *Arabidopsis thaliana*, in combination with the activation domains of yeast *GAL4* and plant *EDLL* and a synthetic promoter harboring the cognate *cis*-regulatory motifs. Chromosomally integrated ATFs and their binding sites (ATF/BSs) resulted in a wide spectrum of inducible transcriptional outputs in *P. pastoris*, ranging from as low as 1- to as high as ∼63-fold induction with only small growth defects. We demonstrated the application of ATF/BSs by generating *P. pastoris* cells that produce β-carotene. Notably, the productivity of β-carotene in *P. pastoris* was ∼4.8-fold higher than that in *S. cerevisiae*, reaching ∼59% of the β-carotene productivity obtained in a *S. cerevisiae* strain optimized for the production of the β–carotene precursor, farnesyl diphosphate, by rewiring the endogenous metabolic pathways using plant-derived ATF/BSs. Our data suggest that plant-derived regulators have a high degree of transferability from *S. cerevisiae* to *P. pastoris*. The plant-derived ATFs, together with their cognate binding sites, powerfully increase the repertoire of transcriptional regulatory modules for the tuning of protein expression levels required in metabolic engineering or synthetic biology in *P. pastoris*.

## Introduction

Owing to the high yield of recombinant protein production, high similarity of the glycosylation pattern to that in mammalian cells (Balamurugan et al., 2007; Gao et al., 2021), and appropriate folding and secretion of recombinant proteins to the extracellular environment (Yang et al., 2013), yeast *Pichia pastoris* (syn. *Komagataella phaffii*) is a broadly used cell factory for the production of various biopharmaceuticals such as insulin (Kjeldsen et al., 1999), hepatitis B surface antigen (Ana Vassileva et al., 2001), human serum albumin (Mallem et al., 2014), and many industrial chemicals including phytase (Tang et al., 2009), lipase (Su et al., 2010), and mannanase (Chen et al., 2020). In contrast to its well−known function as a protein expression system, a limited set of characterized, species-specific synthetic biological tools have been established for *P. pastoris* (Sanjana et al., 2012; Fischer and Glieder, 2019). Therefore, more efforts should be devoted to developing novel synthetic biology tools to establish *P. pastoris* as a robust chassis for the recombinant protein production of biosynthesis of natural products (Gao et al., 2021).

Transcriptional regulators play an important role in heterologous protein production (Hartner et al., 2008; Sanjana et al., 2012). *P. pastoris*, like other eukaryotic organisms, requires a distinct promoter (and terminator) to control the expression of each gene (Tang et al., 2020). Although diverse regulators have been developed in recent years for synthetic biology applications in prokaryotic and eukaryotic cells (Hartner et al., 2008; Bruckner et al., 2015; Naseri et al., 2017; Ji et al., 2019), a limited number of artificial regulators are available for *P. pastoris*. To express heterologous protein and multi-enzymes of a heterologous pathway in *P. pastoris*, its native promoters are commonly used (Massahi and Çalık, 2018; Xu et al., 2018; Prattipati et al., 2020). For example, promoter libraries created by the deletion and duplication of putative transcription factor binding sites within the methanol inducible alcohol oxidase 1 (*AOX1*) promoter (Hartner et al., 2008) or the single point mutation of the constitutive glyceraldehyde-3-phosphate dehydrogenase (*GAPDH*) promoter (Waterham et al., 1997) are being used in *P. pastoris*. However, employing native regulatory elements for heterologous pathway engineering may cause metabolic burden on the cell as the formation of the product competes with cell proliferation and growth (Wu et al., 2016; Naseri et al., 2017) and be undesirable when an orthogonal (minimal interfere with the native cellular processes) and controllable (allowing expression at desired time) system is needed (Naseri and Koffas, 2020). Additionally, the repetitious use of the same regulator in multiple expression cassettes results in genetic instability (Peng et al., 2015). To address the aforementioned challenges, orthogonal transcription factors (TFs) allowing separation of biomass accumulation from the subsequent target molecule production phase, tight control, and tuneable expression of the heterologous gene is desirable (Naseri and Koffas, 2020).

Natural TFs typically contain two functional domains, namely DNA-binding domain (DBD) that target its binding site (BS) within the gene promoter, and activation domain (AD) that activates transcription by interacting with the basal transcription machinery of the cell. Additionally, TFs require nuclear localization signal (NLS) to import to the nucleus of the eucaryotic cell. Recently, pairs of artificial transcription factors (ATFs) and synthetic target promoters [minimal promoter fused to binding site(s) of the ATF were developed as orthogonal regulatory modules. ATFs are based on DBD of various heterologous TF or synthetic DBDs fused to various ADs and NLS. Expression of ATFs is usually controlled by either exogenous chemical inducers [e.g., isopropyl-β-D-thiogalactopyranoside (IPTG)] (Li et al., 2020) or by the light of specific wavelengths (Hartmann et al., 2020), allowing control of the timing of ATF expression and subsequent binding to the synthetic promoter driving expression of a target gene. Until now, inducible ATFs, based on synthetic transcription activator-like effector proteins (TALE) and dead CRISPR-associated protein (dCas9) (Sanjana et al., 2012; Gaj et al., 2013; Machens et al., 2017) have been established for yeast *Saccharomyces cerevisiae* (Naseri et al., 2019). However, little attention has been paid to the implementation of TALE- and dCas9-derived regulators for metabolic engineering mainly due to their relatively large size (> 5.5 kb), the repetitive structure of TALE-based ATFs and low transcriptional activation by dCas9-derived ATFs (Gao et al., 2014). Surprisingly, the small size ATFs based on heterologous DBDs of plant TFs (∼ 2.5–∼ 3 kb) have recently been developed as strong regulators for *S. cerevisiae* (10-fold stronger than the yeast constitutive and strong *TDH3* promoter).

Plant TFs are grouped into diverse families according to conserved motifs that define their DBD. Over 2000 TFs belonging to different families are identified in higher plants, and approximately half of them are plant-specific. Such TFs are potentially well-suited for establishing orthogonal regulators in yeast (or other heterologous) systems. Plant-derived regulators are based on DBD or full-length (consists of DBD and native AD) of various plant-specific TFs, N- or C-terminally fused to various ADs from yeast *GAL4* or plant *EDLL* and NLS and synthetic promoters harboring one to multiple copies of the cognate *cis*-regulatory motifs upstream of a yeast *CYC1* minimal promoter driving a target gene expression (Naseri et al., 2017). The expression of ATF is under the control of an IPTG-inducible yeast *GAL1* promoter. Upon binding ATF to its BS, the downstream gene will be expressed (Naseri et al., 2017). By employing only nine (three weak, three medium, and three strong) plant-derived ATFs and their corresponding BSs, we previously established a combinatorial library of *S. cerevisiae* variants that allow a wide range of gene expression fine-tuning (Naseri et al., 2019). Considering the similarity between *S. cerevisiae* and *P. pastoris*, functional regulators of *S. cerevisiae* are expected to be transferable to *P. pastoris* (Ito et al., 2020). A recent study showed that among the 72 terminators in *S. cerevisiae*, 41 had a high degree of transferability to *P. pastoris* (Ito et al., 2020).

Here, we focused on characterizing plant-derived ATFs for orthogonal gene expression in *P. pastoris*. We integrated a collection of ATF/BSs, covering low to high transcriptional strength, into the *GUT1* (glycerol utilization 1) locus (Vogl et al., 2018) of *P. pastoris*. The data show that plant-derived ATF/BSs had a high degree of transferability from *S. cerevisiae* to *P. pastoris.* Moreover, 30% of the plant-derived regulators of our collection resulted in higher transcriptional output than the constitutive, strong *GAPDH* promoter (Hans R. Waterham et al., 1997) of *P. pastoris.* Additionally, we compared the carotenoid production ability of *P. pastoris* and *S. cerevisiae* capable of fabricating high levels of the β-carotene precursor, farnesyl diphosphate (FPP). Accumulation of FPP in *S. cerevisiae* was achieved by upregulating the expression of ERG20 (FPP synthase), GDH2 (glutamate dehydrogenase), and HMG1 (3-hydroxy-3-methyl-glutaryl-CoA reductase) (Verwaal et al., 2007; Paradise et al., 2008) using inducible, strong plant-derived ATF/BSs, and by deleting *LPP1* (lipid phosphate phosphatase), *DPP1* (diacylglycerol diphosphate phosphatase), and *GDH1* (NADP + glutamate dehydrogenase) (Scalcinati et al., 2012; Trikka et al., 2015). Upon expressing β-carotene pathway genes under the control of plant-derived ATF/BSs, *P. pastoris* produced 70% of the β-carotene obtained from *S. cerevisiae* with optimized background. In conclusion, our data confirm the high transcriptional capacity of plant-derived regulators for bioengineering applications in *P. pastoris*, a suitable host for production of biopharmaceuticals.

## Materials and Methods

### General

Plasmids were constructed using NEBuilder HiFi DNA assembly (New England Biolabs, Frankfurt am Main, Germany) and SLiCE cloning (Zhang et al., 2012). Plasmid and primer sequences are given in Supplementary Tables 1, 2, respectively. PCR amplification of DNA fragments was performed using high-fidelity polymerases: Phusion Polymerase (Thermo Fisher Scientific), Q5 DNA Polymerase (New England Biolabs, Frankfurt am Main, Germany), or PrimeSTAR GXL DNA Polymerase (Takara Bio, Saint-Germain-en-Laye, France) according to the manufacturer’s recommendations. All restriction enzymes were purchased from New England Biolabs (Frankfurt am Main, Germany). Amplified DNA fragments were gel-purified prior to further use. The primers were ordered from Biomers.net (Ulm, Germany). *Escherichia coli* ER2925, NEB 5α, or NEB 10β cells (New England Biolabs) were transformed with the plasmids. Strains were grown in Luria-Bertani medium containing an appropriate selection marker at 37°C (Ampicillin, 100 μg/mL or Kanamycin, 50 μg/mL). The plasmid constructs were confirmed by sequencing (Microsynth Seqlab, Goettingen, Germany).

Genetic transformation of the plasmids or linearized DNA fragments from *S. cerevisiae* was performed using the LiAc/SS carrier DNA/PEG method (Gietz and Schiestl, 2007). The adopted LiAc/SS carrier DNA/PEG method was used to transform *P. pastoris* with plasmids or linearized DNA fragments (Ito et al., 2018). The yeast strains were grown at 30°C in yeast extract peptone dextrose adenine (YPDA)-rich medium [1% (w/v) bacto yeast extract, 2% (w/v) peptone, 2% (w/v) glucose, 0.04% (w/v) adenine hemisulfate, and 2% (w/v) agar for solid medium] or in an appropriate synthetic complete (SC) media [0.67% (w/v) yeast nitrogen base with ammonium sulfate, 2% (w/v) glucose, and 2% (w/v) agar for solid medium] lacking amino acids to allow selection for transformed cells. When required, glucose was replaced by 2% (w/v) glucose. Single copy integration of each linearized plasmid into the *GUT1* site of the *P. pastoris* genome was verified by colony PCR using primers GNPP001 and GNPP002.

### Construction of Plant-Derived ATF and Promoter Pair Clones

To integrate the plasmid into the *GUT1* locus of the *P. pastoris* genome, 1000-bp LHA (primers GNPP003/GNPP004) and 1,000-bp RHA (primers GNPP005/GNPP006) were PCR-amplified from the genomic DNA of the GS115 *P. pastoris* strain. A *Pme*I site was added to the 5′-end of GNPH004 and GNPH005. The expression plasmids, listed in Supplementary Table 3 (Naseri et al., 2017), were digested with *Not*I-HF and *Pme*I and assembled with the LHA and RHA fragments, resulting in the plasmids *pGNPP1* to *pGNPP7* listed in Supplementary Table 4. Subsequently, the *pGNPP1* to *pGNPP7* plasmids were digested with *Aat*II. A synthetic promoter containing a plant TF binding site and a downstream GFP fragment (primers GNPP007/GNPP008) were PCR-amplified from the reporter plasmids listed in Supplementary Table 5 (Naseri et al., 2017). The PCR-amplified fragments were assembled in the digested plasmids to generate integration the plasmids *pGNPP008* to *pGNPP0024* listed in Supplementary Table 6. To construct a positive control plasmid, *pGN005B* was digested with *Not*I-HF and *Bam*HI and subsequently gel-purified to remove a 650-bp fragment. The remaining 6,500-bp fragment was assembled with the PCR-amplified *GUT1* integration locus (primers GNPP004/GNPP009, on pGNPP001) and the *GAPDH1* promoter (primers GNPP010/GNPP011, on yeast GS115 DNA). The generated integration plasmid was named *pGNPP025*. *pGNPP* integration plasmids (see section “Results”) were linearized with *Pme*I (present within the *GU1* homology buffer) and used to transform the JC308 *P. pastoris* strain (Lin Cereghinoa et al., 2001). Integration takes place at the *GUT1* locus of the yeast genome. Positive clones were selected on SC-URA3 medium. The generated *P. pastoris* strains are listed in Table 1. Subsequently, expression of the chimeric TFs was controlled by an IPTG-inducible promoter, and their transactivation capacity was tested against one, two, or four TF binding sites placed upstream of the *CYC1* minimal promoter that controls *GFP* expression.

**TABLE 1:** List of *P. pastoris* strains for characterization of plant-derived ATF/BSs generated in JC308 background (Lin Cereghinoa et al., 2001).

Yeast strain	Relevant genome	Relevant regulator
*yPPGN001*	JC308 + *GUT1*:pGNPP003	*Pro_*mGAL1*_-*NLS-GAL4AD-ANAC102
*yPPGN002*	JC308 + *GUT1*:pGNPP004	*Pro_*mGAL1*_-*NLS-JUB1_*D*__*BD*_-EDLLAD
*yPPGN003*	JC308 + *GUT1*:pGNPP002	*Pro_*mGAL1*_-*NLS-GAL4AD-GRF9
*yPPGN004*	JC308 + *GUT1*:pGNPP024	*Pro_*mGAL1*_-*NLS-EDLLAD-ANAC102-Pro_*CYC*__1m__*in*__–__*ANAC10*__2–4x_
*yPPGN005*	JC308 + *GUT1*:pGNPP008	*Pro_*mGAL1*_-*NLS-EDLLAD-ANAC102-Pro_*CYC*__1m__*in*__–__*ANAC10*__2–2x_
*yPPGN006*	JC308 + *GUT1*:pGNPP009	*Pro_*mGAL1*_-*NLS-GAL4AD-GRF9-Pro_*CYC*__1m__*in*__–__*GRF*__9–1x_
*yPPGN007*	JC308 + *GUT1*:pGNPP010	*Pro_*mGAL1*_-*NLS-GAL4AD-GRF9-Pro_*CYC*__1m__*in*__–__*GRF*__9–2x_
*yPPGN008*	JC308 + *GUT1*:pGNPP011	*Pro_*mGAL1*_-*NLS-GAL4AD-GRF9-Pro_*CYC*__1m__*in*__–__*GRF*__9–4x_
*yPPGN009*	JC308 + *GUT1*:pGNPP012	*Pro_*mGAL1*_-*NLS-GAL4AD-ANAC102-Pro_*CYC*__1m__*in*__–__*ANAC10*__2–1x_
*yPPGN010*	JC308 + *GUT1*:pGNPP014	*Pro_*mGAL1*_-*NLS-GAL4AD-ANAC102-Pro_*CYC*__1m__*in*__–__*ANAC10*__2–4x_
*yPPGN011*	JC308 + *GUT1*:pGNPP015	*Pro_*mGAL1*_-*NLS-JUB1_*D*__*BD*_-EDLLAD-Pro_*CYC*__1m__*in*__–__*JUB*__1–1x_
*yPPGN012*	JC308 + *GUT1*:pGNPP016	*Pro_*mGAL1*_-*NLS-JUB1_*D*__*BD*_-EDLLAD-Pro_*CYC*__1m__*in*__–__*JUB*__1–2x_
*yPPGN013*	JC308 + *GUT1*:pGNPP017	*Pro_*mGAL1*_-*NLS-JUB1_*D*__*BD*_-EDLLAD-Pro_*CYC*__1m__*in*__–__*JUB*__1–4x_
*yPPGN014*	JC308 + *GUT1*:pGNPP018	*Pro_*mGAL1*_-*NLS-GAL4AD-GRF7-Pro_*CYC*__1m__*in*__–__*GRF*__7–1x_
*yPPGN015*	JC308 + *GUT1*:pGNPP019	*Pro_*mGAL1*_-*NLS-GAL4AD-GRF7-Pro_*CYC*__1m__*in*__–__*GRF*__7–2x_
*yPPGN016*	JC308 + *GUT1*:pGNPP020	*Pro_*mGAL1*_-*NLS-GAL4AD-GRF7-Pro_*CYC*__1m__*in*__–__*GRF*__7–4x_
*yPPGN017*	JC308 + *GUT1*:pGNPP013	*Pro_*mGAL1*_-*NLS-GAL4AD-ANAC102-Pro_*CYC*__1m__*in*__–__*ANAC10*__2–2x_
*yPPGN018*	JC308 + *GUT1*:pGNPP021	*Pro_*mGAL1*_-*NLS-EDLLAD-GRF9- Pro_*CYC*__1m__*in*__–__*GRF*__9–1x_
*yPPGN019*	JC308 + *GUT1*:pGNPP022	*Pro_*mGAL1*_-*NLS-EDLLAD-GRF9- Pro_*CYC*__1m__*in*__–__*GRF*__9–2x_
*yPPGN020*	JC308 + *GUT1*:pGNPP023	*Pro_*mGAL1*_-*NLS-EDLLAD-GRF9-1XBS-Pro_*CYC*__1m__*in*__–__*GRF*__9–4x_
*yPPGN021*	JC308 + *GUT1*:pGNPP025	Pro_*GAPDH1*_

### Construction of Plasmids and Donors for the Upregulation of Genes in *S. cerevisiae*

Plasmid *pL0A_0-1* (Hochrein et al., 2017) was digested with *Pme*I and *Sbf*I and assembled with: (i) PCR-amplified ATAF1-derived ATF [primers GNPP012/GNPP013, on *Entry vector X_mGAL1_NLS_GAL4 AD_ATAF1_4X* (Naseri et al., 2019)] and *ERG20* fused to its native terminator (primers GNPP014/GNPP015, on yeast BY4741 DNA) to generate *pGNSC001*; (ii) PCR-amplified JUB1-derived ATF [primers GNPP012/GNPP016, on *Entry vector X_mGAL1_NLS_ GAL4 AD_ JUB1_2X* (Naseri et al., 2019)] and *GDH2* fused to its native terminator (primers GNPP017_1/GNPP017_2, on yeast YPH500 DNA) to generate *pGNSC002*; and (iii) PCR-amplified ANAC102-derived ATF [primers GNPP012/GNPP018, on *Entry vector X_mGAL1_NLS_GAL4 AD_ANAC102_2X* (Naseri et al., 2019)] and *tHMG1* fused to its native terminator (primers GNPP019_1/GNPP019_2, on yeast YPH500 DNA) to generate *pGNSC003*. Donors containing plant-derived ATF-fused *GDH2*-, *tHMG1*-, and *ERG20*-encoding DNA (see section “Results”) were amplified from *pGNSC001*, *pGNSC002*, and *pGNSC003* using primers GNSC020/GNSC021, GNSC022/GNSC023, and GNSC024/GNSC025, respectively. Transformation of strain Gen 0.2 with the three donors and plasmid *pTAJAK105* (Bao et al., 2015; Table 1) allowed integration of the *GDH2*, *tHMG1*, and *ERG20* donors into the *ADE2.a* (Bao et al., 2015), *his3D1* (Bao et al., 2015), and *ura3-52* (Bao et al., 2015) sites of the genome, respectively. Selection for integration was carried out on a medium lacking yeast auxotrophic marker LEU (SC-LEU), since the *LEU2* gene is encoded on *pTAJAK105*. When colonies appeared, the transformation plates were replicated on non-selective plates (YPDA media) to remove the *pTAJAK105* plasmid.

### Construction of Plasmids and Donors for the Inactivation of Genes

The PCR-amplified *SNR52* promoter (primers GNPP026/GNPP027, on *pCRCT*) and *PDC1* terminator (primers GNPP028/GNPP029, on yeast Gen 0.1 DNA; Naseri et al., 2019) were assembled in *Xho*I/*Age*I-digested *pCRCT* (Bao et al., 2015). A sequence containing *gRNA* targeting the *DPP1* gene (392-bp downstream of the start codon), *tracrRNA*, and the *SUP4* terminator was introduced after the *SNR52* promoter through the primer sequences. The generated plasmid was named *pGNSC004*.

The PCR-amplified *CYC1* terminator (primers GNPP030/GNPP031, on *p426-SNR52p-gRNA.CAN1.Y-SUP4t*; Addgene #43803) was assembled in *Sma*I/*Sal*I-digested *pGNSC004*, introducing a *gRNA* targeting *LPP1* (429-bp downstream of the start codon) and *tracrRNA* after the *SNR52* promoter. The resulting plasmid was named *pGNSC005*.

*Sal*I/*Asc*I*-*digested *pGNSC004* was assembled with double-stranded oligonucleotides (primers GNPP032/GNPP033), introducing a *gRNA* targeting the *GDH1* gene (658-bp downstream of the start codon). The generated plasmid was named *pGNSC006*.

Subsequently, *Not*I-digested *pGNSC004* was assembled with the PCR-amplified *LPP1 gRNA* encoding cassette (primers GNPP034/GNPP035, on *pGNSC005*). The generated plasmid was called *pGNSC007*.

Subsequently, *Not*I/*Pac*I-digested *pGNSC007* was assembled with the PCR-amplified *GDH1-gRNA* encoding cassette (primers GNPP036/GNPP037, on *pGNSC006*). The generated plasmid was called *pGNSC008*.

Moreover, three donor DNAs, allowing a frameshift mutation in *DPP1*, *LPP1*, and *GDH1*, were constructed by annealing single-strand oligonucleotides (primers GNPP038/GNPP039, GNPP040/GNPP041, and GNPP042/GNPP043, respectively). Each donor contains a 50-bp homology arm in the up- and downstream targeting sequence of the gene. Three donors, together with *pGNSC008* (expressing *gRNAs*, *tracer RNAs*, and iCas9 protein), were used to transform yeast cells. Selection for integration was carried out on a medium lacking the yeast auxotrophic marker URA (SC-URA), since the *URA3* gene is encoded on *pGNSC008*. When colonies appeared, the transformation plates were replicated on non-selective plates (YPDA media) to remove the *pGNSC008* plasmid.

### FPP-Producing *S. cerevisiae* Strains

We combined the previously reported HI-CRISPR system (Bao et al., 2015) and three plant-derived ATF/BSs, namely NLS-GAL4AD-JUB1-2X (Naseri et al., 2019), NLS-GAL4AD-ANAC102-2X (Naseri et al., 2019), and NLS-GAL4AD-ATAF1-4X (Naseri et al., 2019), to overexpress yeast genes, *GDH2*, *tHMG1*, and *ERG20*, respectively. To construct a strain overexpressing GDH2, tHMG1, and ERG20, previously characterized integration sites, shown to exhibit a high integration efficiency, were targeted (Bao et al., 2015). The *GDH2*, *tHMG1*, and *ERG20* donors were integrated, respectively, at genomic *ADE2*.a, *his3D1*, and *ura3-52* loci of yeast Gen 0.2, where each donor is integrated into a single locus (Figure 5A). Each donor contains an IPTG-inducible ATF/BS upstream of a *CDS* and ends with a yeast terminator. The 50-bp overhang sequences up- and downstream of each donor allow its integration into pre-designed genomic loci.

**FIGURE 1:**
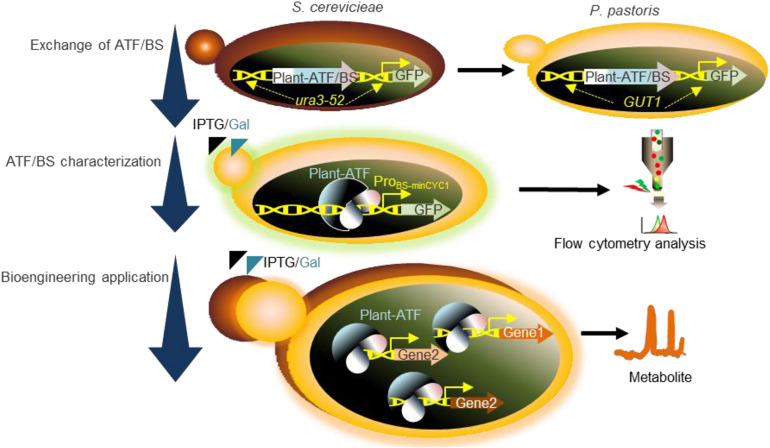
Schematic overview of the workflow used in the present study. Exchange of ATF/BSs: our plant-derived GFP-ATF/BSs deriving GFP expression, previously characterized following integration into the *ura3-52* locus of *S. cerevisiae* (Naseri et al., 2017), were inserted into the *GUT1* locus of *P. pastoris*. ATF/BS characterization: following the addition of IPTG and galactose (inducers), GFP expression was determined by flow cytometry. Bioengineering application: characterized ATF/BSs were employed to tune the protein expression levels required for metabolic engineering in *P. pastoris*. ATF/BS, artificial transcription factor and its corresponding binding site; GUT1, glycerol utilization 1; Pro_*BS*__–__*minCYC1*_, synthetic promoter containing the binding sites of plant TFs upstream of the minimal *CYC1* promoter.

**FIGURE 2:**
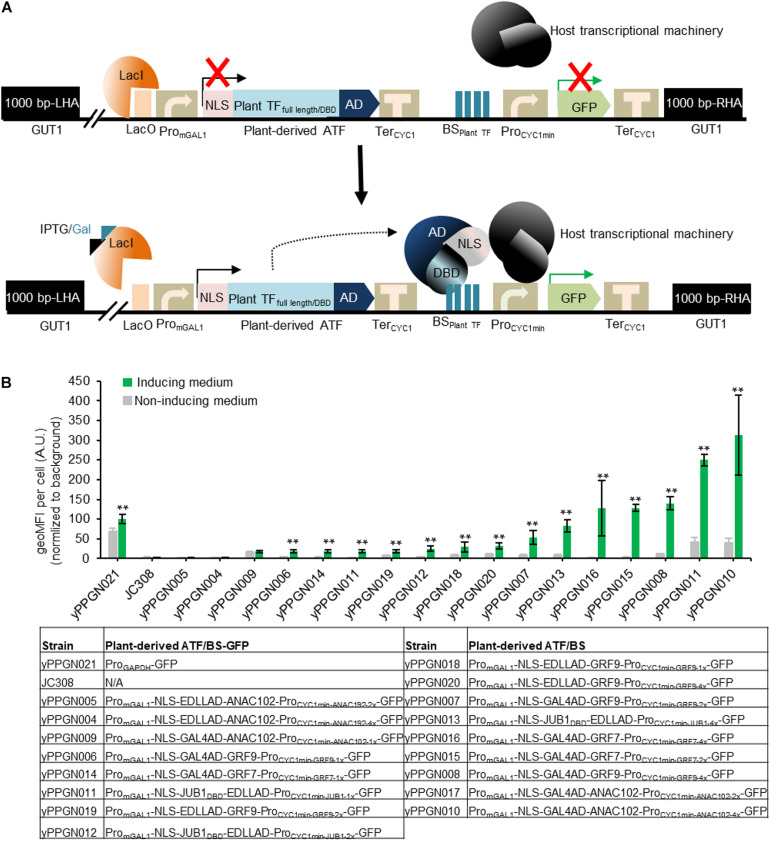
Library of genome-integrated, plant-based ATF/BSs in *P. pastoris*. **(A)** Schematic overview of plant-derived ATFs and BSs used in the present study. The ATF cassette contains an *mGAL1* promoter with a lacO site at the 5′ end (induced by IPTG and galactose), NLS, and full-length plant TFs or their DBDs fused in different combinations with GAL4AD or EDLLAD and *Ter*_*CYC1*_. The transactivation capacity of the ATFs was tested against the TF BSs inserted as one (1x), two (2x), or (4x) copies upstream of the *CYC1* minimal promoter driving GFP reporter expression. The 1,000-bp LHA and RHA allow integration of the regulator cassette into the *GUT1* sites of the genome. The expression of ATFs was controlled by an IPTG-inducible *GAL1* promoter. Constitutive expression of the repressor (LacI) inhibits the expression of plant-derived ATFs, while the addition of inducers (IPTG and galactose) results in ATF expression. Binding of the ATF to its cognate BS within the *CYC1* minimal promoter drives GFP expression. Fluorescence output is measured in the absence and presence of inducer. AD, activation domain; ATF, artificial transcription factor; BS_*plant TF*_, binding site of the plant transcription factor; BS, binding site; DBD, DNA binding domain; GFP, green fluorescent protein; IPTG, isopropyl-β-D-thiogalactopyranoside; GUT1, glycerol utilization 1; LHA, left homology arm; NLS, nucleus localization signal; Pro_*minCYC1*_, minimal *CYC1* promoter; *Pro*_*mGAL*__1_, modified GAL1 promoter carry *LacO* site; LacI, Lac repressor; Ter_*CYC1*_, *CYC1* terminator; RHA, right homology arm. **(B)** Transcriptional output of plant-derived ATF/BSs in *P. pastoris*. The GFP output was tested in the absence and presence of IPTG and galactose. The types of ATF used to generate yPPGN004—yPPGN020 strains are listed in the table. *P. pastoris* strain yPPGN021 was used as a positive control, where the GFP expression is controlled by *GAPDH* promoter allowing constitutive expression of GFP in both inducing- and non-inducing medium. EDLLAD, activation domain from plant; GAL4, GAL4 activation domain of yeast; GRF7 and GRF9, Growth-Regulating Factor 7 and 9; JUB1, NAC TF JUNGBRUNNEN1; ANAC102, A NAC TF 102; VP64AD, viral activation domain. Gray, non-induction medium; green, induction medium. Data are expressed as the geometric mean ± SD of the fluorescence intensity obtained from three cultures, each derived from an independent yeast colony and determined in three technical replicates. Data are normalized to the average geoMFI of the background fluorescence obtained from the wild-type JC308 strain (Lin Cereghinoa et al., 2001). Asterisks indicate a statistically significant difference from the non-induction medium (Student’s *t-*test; **p < 0.01). AU, arbitrary units; geoMFI, geometric mean fluorescence. The full data are shown in Supplementary Data 1.

**FIGURE 3:**
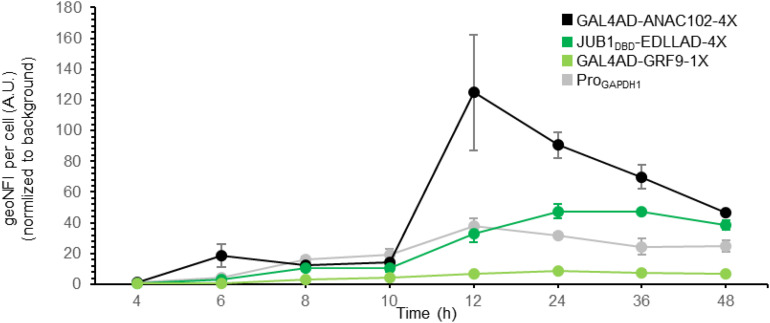
Time course of plant-derived ATF/BS-dependent reporter gene induction. Time course of reporter gene induction, resulting from plant-derived ATFs consisting of GAL4AD and ANAC102 (black), the DBD of JUB1 and EDLLAD (green), or GAL4AD and GRF9 (light green) in combination with “4,” “4,” or “1” copies of their BS, respectively, was compared with the fluorescent output of the constitutive *GAPDH* promoter (gray). The output of GFP was measured by flow cytometry after 4, 6, 8, 10, 12, 24, 36, and 48 h. The samples were harvested at each time point and analyzed for GFP expression. At each time point, the medium was replaced with the fresh induction medium. EDLLAD, activation domain from plant; GAL4AD, GAL4 activation domain of yeast; GRF9, Growth-Regulating Factor 9; JUB1, NAC TF JUNGBRUNNEN1; ANAC102, A NAC TF 102; DBD, DNA binding site; Pro, promoter. Data are expressed as the geometric mean ± SD of the fluorescence intensity obtained from three cultures, each derived from an independent yeast colony and determined in two technical replicates. Data are normalized to the average geoMFI of the background fluorescence obtained from wild-type JC308. AU, arbitrary units; geoMFI, geometric mean fluorescence. The full data are shown in Supplementary Data 2.

**FIGURE 4:**
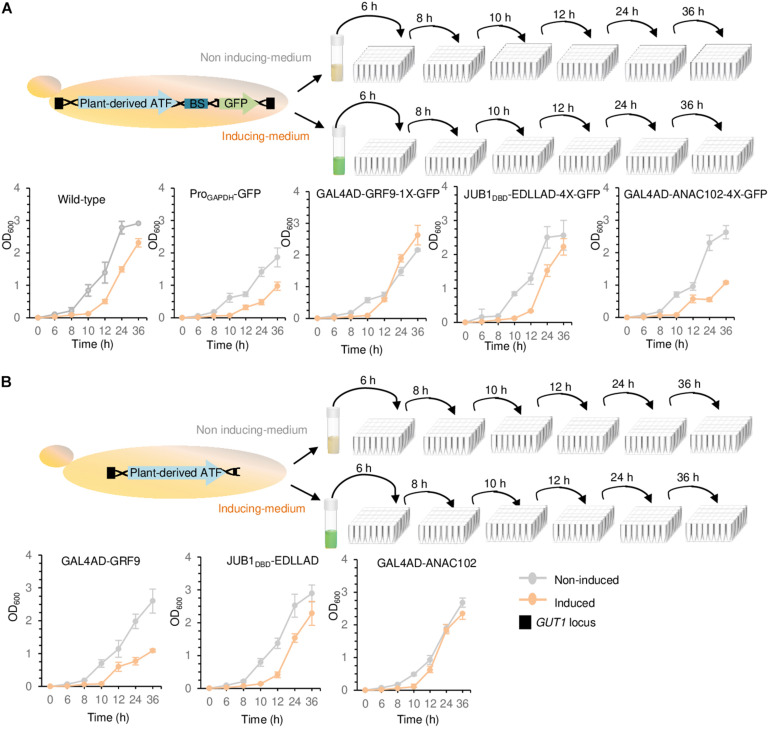
Growth assays. **(A)** Growth assay of yeast cells containing chromosomally integrated driver and reporter modules. **(B)** Growth assay of yeast cells containing chromosomally integrated driver modules. The strains containing either the integrated driver/reporter or driver modules were inoculated in induction (orange line) and non-induction (gray line) medium, followed by OD_600_ measurement at 0, 6, 8, 10, 12, 24, and 36 h. Wild-type JC308 was used as a control. “1X” and “4X” indicate one or four copies of the TF binding site, respectively. To simplify the figure, the modified *GAL1* promoter and *CYC1* terminator located upstream and downstream of plant-derived ATFs, NLS, and the minimal *CYC1* promoter located upstream of GFP are not shown. EDLLAD, activation domain from plant; GAL4AD, GAL4 activation domain of yeast; GRF9, Growth-Regulating Factor 9; JUB1, NAC TF JUNGBRUNNEN1; ANAC102, A NAC TF 102; DBD, DNA binding site; Pro, promoter. Data are expressed as the mean OD_600_ values ± SD obtained from three cultures, each derived from an independent yeast colony and determined in three technical replicates. OD_600_, optical density at 600 nm. The full data are shown in Supplementary Data 3.

**FIGURE 5:**
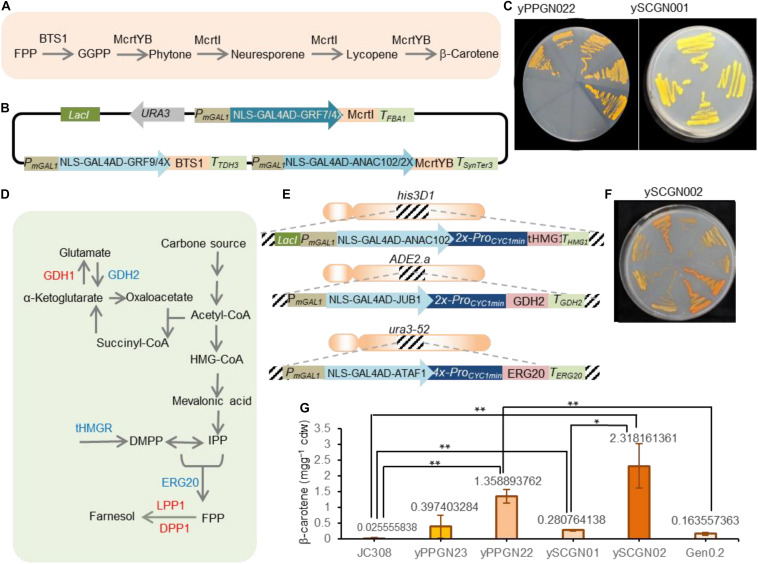
Production of β-carotene in yeast strains. **(A)** Scheme showing the β-carotene production from FPP precursor. BTS1, geranylgeranyl diphosphate synthase; FPP, farnesyl diphosphate; GGPP, geranylgeranyl diphosphate; McrtYB, optimized phytoene synthase/lycopene cyclase; McrtI, phytoene desaturase. **(B)** Scheme showing the β-carotene-encoding plasmid. The pCAROTENE plasmid was constructed as described by Naseri et al. (2019). *McrtI*, *McrtYB*, and *BTS1* genes are under the control of ATF/BSs NLS-GAL4AD-GRF9-4X, NLS-GAL4AD-GRF7-4X, and NLS-GAL4AD-ANAC102-4X. The *ATF/BS-McrtI*, *-McrtYB*-, and *-BTS1* modules are flanked by the IPTG inducible modified *GAL1* promoter. Additionally, the *ATF/BS-McrtI* module encodes the *LacI* repressor. Selection on SC-URA media allows screening for successful plasmid integration. To simplify the figure, the *CYC1* terminator located downstream of ATF is not shown. AD, activation domain; ATF, artificial transcription factor; BS_*plant TF*_, binding site of the plant TF; DBD, DNA binding domain; IPTG, isopropyl-β-D-thiogalactopyranoside; NLS, nucleus localization signal; Pro_*minCYC1*_, *CYC1* minimal promoter; Ter, terminator. **(C)** Representative plate of the constructed β-carotene producing strains. The pCAROTENE plasmid was transformed to *P. pastoris* and *S. cerevisiae* to generate the yPPGN022 and ySCGN01, respectively. Upon adding IPTG, plant-derived ATFs were expressed and β-carotene was produced. Colors of carotenoid-producing yeast strains in YPDA media for the yPPGN022 and ySCGN01 strains. **(D)** Scheme illustration of FPP precursor production in yeast. To redirect yeast metabolisms toward the FPP production, GDH2, ERG20, and tHMG1 overexpression (blue), deleted or inactive *DPP1*, *LPP1* and *GDH1* (red) are needed. ERG20, FPP synthase; DPP1 and LPP1, lipid phosphate phosphatases; tHMG1, truncated HMG-CoA reductase, GDH1, NADP + -glutamate dehydrogenase; GDH2, NAD + -dependent glutamate dehydrogenase; HMG-CoA, 3-hydroxy-3methylglutaryl-CoA; IPP, isopentenyl diphosphate; DMAPP: dimethylallyl diphosphate. **(E)** Scheme representing overexpressing donors for the enhanced production of FPP. ATFs *NLS-GAL4AD-ANAC102-2X*, *NLS- GAL4AD-JUB1-2X*, and *NLS-GAL4AD-ATAF1-4X* are fused to *tHMG1-T_*HMG1*_*, *GDH2-T_*GDH2*_*, and *ERG20-T_*ERG20*_*. The *tHMG1*, *GDH2*, and *ERG20* donors are flanked by 50-bp homology arms to integrate into the *his3D1*, *ADE2.a*, and *ura3-52* loci, respectively. In each donor, a modified *GAL1* promoter is located upstream of the plant-derived ATF. Additionally, the *tHMG1* donor encodes LacI. The BS of ATFs is placed upstream of the *CYC1* minimal promoter to drive gene expression. NLS, nuclear localization signal; JUB1, plant JUNGBRUNNEN1 transcription factor; GAL4AD, yeast GAL4 activation domain; ANAC102, NAC domain containing protein 102; ATAF1, *Arabidopsis thaliana* Activating Factor 1; “2X”, and “4X” indicate two, or four copies of the plant TF binding site, respectively. **(F)** Colors of the carotenoid-producing ySCGN02 *S. cerevisiae* strain (optimized for FPP production) in YPDA media. **(G)** SFC analysis of the carotenoid content of yeast strains. Values represent the mean ± SD of three independent colonies after 3 days of cultivation. *P. pastoris* strains yPPGN023 containing pCAROTENE-PGAP plasmid and J308 were used as a positive and negative controls, while Gen 0.2 was used as a negative *S. cerevisiae* control. Asterisks indicate a statistically significant difference (Student’s *t-*test; *p < 0.05; **p < 0.01). Cdw, cell dry weight. Full data are shown in Supplementary Data 4.

For inactivation of the *GDH1*, *DPP1*, and *LPP1* genes, three donors, together with *pCRCT-GDH1-DPP1-LPP1* (expressing *gRNAs*, *tracer RNAs*, and iCas9 protein), were used to transform yeast cells, where each donor is integrated into a single locus. Each donor contains the 50-bp overhang sequences up- and downstream of its integration into target genomic sites, and incorporates an 8-bp deletion included within the target gene, thus introducing a frameshift mutation. The ySCGN0.1 or ySCGN0.2 strains were obtained by integrating overexpression or inactivation donors into the yeast genome, respectively. The ySCGN1.2 strain harbors inactivated genes, *lpp1*, *dpp1*, and *gdh1*, in addition to overexpressed *GDH2*, *tHMG1*, and *ERG20*.

### Induction Experiments, Flow Cytometry, and Data Analysis

To determine the effect of plant-derived ATF/BS on GFP fluorescence output, single colonies of yeast reporter strains were inoculated into 250 μl SC-URA medium containing 2% glucose in 96-well deep-well plates. Plates were incubated for 16 h at 30°C in a rotary shaker at 230 rpm. The precultures were used to inoculate main cultures in 250 μl YPDA containing 2% glucose (non-induction medium) or YPDA containing 2% galactose and 20 mM IPTG (induction medium) to an OD_600_ ∼0.1. Each strain was inoculated in three technical replicates per experiment. Cells were grown at 30°C for 12 h in a rotary shaker at 230 rpm. Samples were harvested after 4, 6, 8, 10, 12, 24, 36, and 48 h (see section “Results”) and analyzed using a BD FACSCalibur Flow Cytometer (BD Biosciences). At each time point, the medium was replaced with the fresh induction medium. GFP fluorescence values were obtained from a minimum of 10,000 cells in each sample. The geometric mean of the GFP fluorescence per cell was calculated using FlowJo Software.

### Growth Assays

Growth assays were done similarly to induction experiments, except that experimental cultures were inoculated to an OD_600_ of ∼0.05 and grown at 30°C and 230 rpm in a rotary shaker. OD_600_ was measured after 6, 8, 10, 12, 24, and 36 h. At each time point, the medium was removed and the fresh induction medium was added to each well.

### Positive Control for β-Carotene Production

The β-carotene pathway genes *McrtI*-, *McrtYB*-, and *BTS1*-CDSs under the control of inducible ATFs *NLS-GAL4AD-GRF7/4X*, *NLS-GAL4AD-ANAC102/2X*, and *NLS-GAL4AD-GRF9/4X* were previously assembled in Destination vector I to generate plasmid pCAROTENE (see COMPASS protocol; Naseri et al., 2019). Destination vector I was digested with *Eco*RI/*Sal*I and used in a two-way assembly reaction using PCR-amplified Pro_*GAPDH*_ sequence (PROGAPMCRTI_for/PROGAPMCRTI_rev, on genomic DNA of the GS115 *P. pastoris*) and *McrtI* CDS fussed to *Ter*_*FBA1*_ (MCRTIPOS_for/MCRTIPOS_rev, on Entry vector X-McrtI; Naseri et al., 2019) to generate pMCRTI_PGAP. Next, *Not*I-digested pCOMPASS32 was used in an assembly reaction using PCR-amplified *Pro*_*GAPDH*_ sequence (PROGAPMCRTI_for/PROGAPMCRTI_rev, on genomic DNA of the GS115 *P. pastoris*) and *BTS1* CDS fussed to *Ter*_*TDH3*_ (BTSPOS_for/BTSPOS_rev, on Entry vector X-BTS1; Naseri et al., 2019). *Not*I-digested pCOMPASS33 was used in an assembly reaction using PCR-amplified *Pro*_*GAPDH*_ sequence (PROGAPMCRTI_for/PROGAPMCRTI_rev, on genomic DNA of the GS115 *P. pastoris*) and *McrtYB* CDS fussed to Ter_*SYN*__3_ (MCRTYBPOS_for/MCRTYBPOS_rev, on Entry vector X-McrtYB; Naseri et al., 2019) to generate pMCRTYB_PGAP. *I*-*Ceu*I- digested pMCRTI_PGAP was used in an assembly reaction using PCR-amplified *Pro*_*GAPDH*_-*BTS1*-*Ter*_*TDH3*_ (PGAP_BTS1_for/PGAP_BTS1_rev, on pBTS1-PGAP). The resulting plasmid was called pMCRTI-BTS1_PGAP. Then, *Fse*I/*I*-*Sce*I-digested pMCRTI-BTS1_PGAP was used in an assembly reaction using PCR-amplified *Pro*_*GAPDH*_–*McrtYB*-*Ter*_*SYN3*_ (PGAP_MCRTYB_for/PGAP_MCRTYB_rev, on pMCRTYB-PGAP). The resulting plasmid was called pCAROTENE-PGAP.

### β-Carotene Production

The yeast strains transformed with pCAROTENE plasmid (Naseri et al., 2019) were plated on SC-URA [supplemented with 2% (w/v) glucose]. Cells were grown at 30°C for 3–4 days. The cells were then plated on induction SC-URA medium plates containing 20 mM IPTG and 2% (w/v) galactose and 1 M sorbitol. Cells were grown at 30°C for 3–4 days. Subsequently, colony PCR was performed, followed by sequencing. Three independent colonies were chosen for β-carotene analysis by SFC. Yeast colonies were inoculated into 4 mL non-induction medium SC-URA [supplemented with 2% (w/v) glucose] and grown for 18–24 h at 30°C and 230 rpm in a rotary shaker. Subsequently, the pre-cultures were used to inoculate main cultures (50 mL) in induction medium [YPDA with the 20 mM IPTG, 2% (w/v) galactose, and 1 M sorbitol]. All shake-flask cultures were inoculated from pre-cultures to an initial OD_600_ of 0.1. Cells were then grown in a 500 mL shake flask for 3 days at 30°C and 230 rpm to saturation.

### Supercritical Fluid Chromatography (SFC)

Carotenoid extraction from saturated culture was carried out according to the acetone extraction method with some modifications (Lian et al., 2017). The stationary phase yeast cells were collected by centrifugation at 13,000 × g for 1 min and cell precipitates were resuspended in 1 mL of 3N HCl, boiled for 5 min, and then cooled in an ice-bath for 5 min. The lysed cells were washed with ddH_2_O and resuspended in 400 μL acetone to extract β-carotene. The cell debris was removed by centrifugation. The acetone supernatant was collected in 2-mL microcentrifuge tubes. This extraction procedure was repeated until the cell pellet was white.

SFC analyses were carried out in triplicates. Carotenoids were separated by SFC Waters^TM^ in 5 min using packed Silica column 4.6 × 150 mm (5 μm) with carbon dioxide modified with 2% (v/v) Tetrahydrofuran (THF) as a mobile phase. The column temperature was set to 40°C and the backpressure was 100 bar, at a flow rate of 1 mL/min. The results were detected using a photodiode array (PDA) detector at 510 nm and analyzed with ChromScope Software.

## Results

### Plant-Derived ATF/BS Units

A central aim of our work was to establish inducible, heterologous regulators for future use in synthetic biology dealing with *P. pastoris*. To this end, 17 ATF/BS combinations from two Arabidopsis TF families, namely: Growth-Regulating Factor 7 (GRF7) (Kim et al., 2012), GRF9 (Kim et al., 2003), the NAC TFs JUNGBRUNNEN1 (JUB1) (Wu et al., 2012), and ANAC102 (Christianson et al., 2009), were selected from our library of plant-derived ATF/BSs developed for *S. cerevisiae* (Naseri et al., 2017). The selected ATF/BS combinations resulted in weak (NLS-EDLLAD-GRF9/1x, NLS-JUB1_*D*__*BD*_-EDLLAD/1x, NLS-J UB1_*D*__*BD*_-EDLLAD/2x, NLS-GAL4AD-GRF7/1x, NLS-EDLLAD-GRF9/2x, NLS-GAL4AD-GRF9/1x, NLS-GAL4AD-GRF9/2x; 0–400 AU), medium (NLS-GAL4AD-GRF9/4x, NLS-GAL4AD-ANAC102/1x, NLS-EDLLAD-GRF9/4x, NLS-GAL4AD-GR F7/2x, NLS-JUB1_*D*__*BD*_-EDLLAD/1x, NLS-GAL4AD-GRF7/4x; 4 00–1,200 AU), and strong (NLS-GAL4AD-ANAC102/2x, N LS-GAL4AD-ANAC102/4x, NLS-EDLLAD-ANAC102/2x, NLS-EDLL-GAL4AD-ANAC102/4x, NLS-EDLLAD-ANAC102/2x, NLS-EDLLAD-ANAC102/4x; 1,200–8,000 AU) transcriptional outputs and low basal expression, upon integration into the *ura3-52* locus of the genome for transcriptional level characterization (Naseri et al., 2017). The selected ATF/BSs spanned a transcriptional activity (determined by GFP expression levels) ranging from ∼0.1- to ∼9-fold as compared with the strong *TDH3* promoter in *S. cerevisiae* (Supplementary Figure 1; Naseri et al., 2017). To ensure a fair comparison of the ATF/BSs in *P. pastoris*, each of the regulator cassettes was inserted into the *GUT1* locus, which leads to high specific integration rates by native homologous recombination and low random genomic integration frequency (Schwarzhans et al., 2016; Vogl et al., 2018). Following selection of a single GFP-transgene, the transcriptional output of plant-derived ATF/BSs was assessed using flow cytometry. The characterized ATF/BSs were subsequently used for metabolic engineering applications by producing β-carotene in *P. pastoris* (Figure 1).

### A Wide Range of Transcriptional Outputs for Plant-Derived ATF/BSs in *P. pastoris*

Plant-derived ATFs, consisting of different combinations of full-length plant TFs or their DBDs, heterologous ADs, and different numbers of the respective *cis*-regulatory motifs located upstream of the yeast *CYC1* minimal promoter, were integrated into the *GUT1* locus in the *P. pastoris* genome. GUT1 is an essential enzyme for glycerol metabolism. Strains with deleted *gut1* (due to integration of ATF/BS cassettes within *GUT1* locus) cannot grow on glycerol (Vogl et al., 2018), while growth on glucose or galactose is not reduced. Following the addition of inducer (20 mM IPTG and 2% galactose), ATFs are expressed and target their BSs within the synthetic promoter (upstream of the yeast *CYC1* minimal promoter), resulting in GFP expression (Figure 2A). As shown in Figure 2B, we achieved a wide spectrum of transcriptional outputs for plant-derived ATF/BSs. Of note, five of the ATFs (30%) resulted in up to a ∼3.13-fold higher GFP reporter output than the yPPGN021 control strain expressing the native *GAPDH* promoter (Figure 2B, strains yPPGN016, yPPGN008, yPPGN011, and yPPGN010). We observed the strongest transcriptional activation capacity for NAC TF-derived ATF NLS- GAL4AD-ANAC102 in combination with 2 or 4 copies of its BS (Figure 2B, yPPGN005 and yPPGN004). Surprisingly, the EDLLAD-ANAC102 combination resulted in a low transcriptional output in *P. pastoris* despite being categorized as a strong regulator in *S. cerevisiae* (Naseri et al., 2017). A combination of EDLLAD and plant TFs typically results in a high expression level of the reporter gene in *S. cerevisiae* (Naseri et al., 2017); however, EDLLAD-derived ATFs (yPPGN04, yPPGN04, yPPGN18-20) resulted in low-to-medium expression of the reporter gene in all our tested drivers/reporters in *P. pastoris*.

### Characterization of Plant-Derived ATF/BSs in *P. pastoris*

We previously showed that by employing only nine inducible plant-derived ATF/BSs, a library of *S. cerevisiae* variants with a million members was established, allowing the fine-tuning of naringenin and β-carotene over a wide range (Naseri et al., 2019). Hence, here we aimed to further prove the transcriptional activation capacity of our plant-derived ATF/BSs for metabolic engineering purposes in *P. pastoris*, rather than extending the size of our library. For full exploitation of the plant-derived ATF/BSs, we further characterized the transcriptional output of a weak GRF9-, medium JUB1_*D*__*BD*_-, and strong ANAC102-derived ATF/BS (yPPGN06, yPPGN13, and yPPGN10, respectively) in comparison with the constitutive *GAPDH* promoter (Waterham et al., 1997) in induction medium over time (Figure 3). Cells were grown in 20 mL induction medium in 100-mL flasks. The output of GFP was measured by flow cytometry over a 48-h time-course.

JUB1_*D*__*BD*_- and ANAC102-derived ATF/BS allow tuning GFP expression from low to high responses, compared the *GAPDH* promoter of *P. pastoris*. The GFP fluorescent output of the JUB1_*D*__*BD*_-derived ATF/BS (Figure 3, green line) reached to the level of GFP was expressed from the *GAPDH* promoter (Figure 3, gray line) 12 h post-induction and was stable for at least 24 h. In contrast, the strong ANAC102-derived ATF/BS (Figure 3, black line) mediated a much higher expression output 12 h post-induction as compared with the *GAPDH* promoter. However, the reporter expression decreased dramatically and reached the level derived from the *GAPDH* promoter after 48 h. Growing *P. pastoris* to a high density is of great benefit when producing a heterologous protein (Karbalaei et al., 2020); however, a high level of oxygen is required to achieve a high yield, which demands a culture vessel to deliver a sufficient amount of oxygen to the yeast (Eck et al., 2018). Due to the inadequate essential nutrients (amino acids, carbohydrates, minerals, vitamins), hormones, growth factors, and change of the physicochemical environment (pH, osmotic pressure), as well as limited oxygen availability (Krause et al., 2016), the reporter expression derived from plant-derived ATFs could be negatively affected in our study. This effect might be more severe in the case of strong ANAC102-derived ATF In contrast, we observed that the weak GRF9-derived ATF/BS (Figure 3, light green line) resulted in a stable expression level of the reporter over time.

### Growth Effects of Plant-Derived ATF and Promoter Pairs in *P. pastoris*

We studied the effect of a weak GRF9-, medium JUB1_*D*__*BD*_-, and strong ANAC102-derived driver/reporter (yPPGN06, yPPGN13, and yPPGN10, respectively) on cell growth. Following integration of the ATF/BS-GFP modules into the *GUT1* locus of the genome, cell growth in induction (Figure 4A, solid orange line) and non-induction (Figure 4A, solid gray line) medium was assessed over 36 h. Cells expressing chromosomally integrated GFP under the control of the constitutive *GAPDH* promoter were used as a positive control. The growth curves of wild-type JC308 cells (not containing ATF) in induction (dashed orange line) and non-induction (dashed gray line) medium are included in all growth plots. The growth of wild-type JC308 was reduced in the induction medium as compared with that in the non-induction medium, demonstrating a negative effect of galactose on cell growth. The strain expressing the strong driver/reporter GAL4AD-ANAC102-2X-GFP (in induction medium) showed similar growth defects to the positive control strain (constitutively expressing the GFP reporter under the control of the *GAPDH* promoter). In contrast, the strains expressing the medium JUB1_*D*__*BD*_-EDLLAD-4X-GFP and weak GAL4AD-GRF9-1X-GFP drivers/reporters displayed only minor growth reduction as compared with the wild-type control strain. Moreover, our data reveal that when the ATFs are present within the genome but are not expressed (non-induction medium), yeast growth was only slightly affected. To assess whether the growth penalty is related to the high level of GFP production, strains expressing only the driver modules GAL4AD-ANAC102. JUB1_*D*__*BD*_-EDLLAD, or GAL4AD-GRF9 (yPPGN01, yPPGN02, and yPPGN03 strains, respectively) (no reporter module) were generated (Figure 4B). JUB1_*D*__*BD*_-EDLLAD strain showed similar growth defects to the JUB1_*D*__*BD*_-EDLLAD-4X-GFP expressing both the driver and reporter, suggesting that cell growth was slightly affected by the expression of fluorescent reporter protein derived from the binding sites of medium JUB1-derived ATF/BS. We observed that the expression of the strong ANAC102-derived ATF driver slightly affects the cell growth (Figure 4B, orange line), while the presence of the ANAC102 driver/reporter module resulted in a growth defect similar to constitutive Pro*_*GAPDH*_* (Figure 4A, orange line). Moreover, the cells expressing ATFs showed a minor growth defect in the non-induction medium, compared to the cells expressing Pro*_*GAPDH*_* (Figure 4B, gray line), emphasizing that the use of inducible promoters for the expression of ATFs may minimize the consumption of energy and nutrient resources and maximize biomass production capacity prior to the production phase.

### Plant-Derived ATF/BS Capacity for β-Carotene Production in *P. pastoris* and *S. cerevisiae*

We sought to confirm the transcriptional ability of plant-derived ATF/BSs for metabolic engineering applications in *P. pastoris* by testing a well-known phenotype. Although *P. pastoris* is a non-carotenogenic yeast, it has been used for the production of carotenoids (Araya-Garay et al., 2012). To convert FPP precursor to β-carotene, geranylgeranyl diphosphate synthase (BTS1), phytoene synthase/lycopene cyclase (McrtYB), and phytoene desaturaseare (McrtI) enzymes are needed (Figure 5A). Here, we transformed yeast *P. pastoris* cells (wild-type JC308 background) with the episomal *pCAROTENE* plasmid (Naseri et al., 2019; Figure 5B) encoding the *McrtI*-, *McrtYB*-, and *BTS1*-CDS of the β-carotene pathway under the control of the inducible ATF/BSs, NLS-GAL4AD-GRF9-4X, NLS-GAL4AD-GRF7-4X, and NLS-GAL4AD-ANAC102-4X (Naseri et al., 2017, ∼1.27—∼3.13-fold stronger than *Pro*_*GAPDH*_) to generate the yPPGN022 strain (Table 2). Selection for the assembly was carried out on a medium lacking the yeast auxotrophic marker, URA3 (the selection marker gene is encoded on the plasmid carrying β-carotene pathway genes). Following induction of ATF expression, orange colonies were observed on the yeast plate (Figure 5C for yPPGN022). After transformation with the *pCAROTENE* plasmid, we observed stronger β-carotene accumulation in the yPPGN022 *P. pastoris* strain than that in the *S. cerevisiae* strain (Table 1 and Figure 5C for ySCGN01) [ySCGN01 has wild-type Gen 0.2 *S. cerevisiae* background (Naseri et al., 2019)] in induction medium (production phase). To further compare the β-carotene productivity of the two hosts, *P. pastoris* and *S. cerevisiae*, we constructed an optimized *S. cerevisiae* cell factory for isoprenoid production by redirecting carbon flux toward the production of the isoprenoid precursor, FPP (Figure 5D; Scalcinati et al., 2012; Lopez et al., 2015). Using CRISPR/Cas9-mediated one-step double-strand breaks (Bao et al., 2015): (i) three modules expressing IPTG-inducible super-strong plant-derived ATF/BSs were integrated into the genome to upregulate the expression of *GDH2*, *tHMG1*, and *ERG20* (Figure 5E and Table 2 for ySCGN0.1); and (ii) *DPP1*, *LPP1*, and *GDH1* were inactivated (Table 2 for ySCGN0.2). Transformation of the ySCGN1.2 strain, carrying both upregulation and inactivation groups (Table 2), with the *pCAROTENE* plasmid produced yeast colonies with a more intense color (Figure 5F and Table 2 for ySCGN02) as compared with the *P. pastoris* yPPGN022 strain. We selected three colonies from each strain for the quantitative determination of β-carotene content by supercritical fluid chromatography (SFC).

**TABLE 2:** List of yeast strains used for β-carotene production in the present study.

Yeast strain	Relevant genome	Origin
JC308	NRRL-Y11430 + ade1, arg4, his4, ura3	Lin Cereghinoa et al., 2001
yPPGN022	JC308 + *pCAROTENE*	This study
yPPGN023	JC308 + *pCAROTENE-PGAP*	This study
IMX672	*MAT a*, *MAL2-8^*c*^*, *SUC2*, *his3D1*, *leu2-3_112*, *ura3-52*, *trp1-289, can1D:Cas9-natNT2*	Euroscarf, #Y40595
Gen0.2	IMX672 + *lys2a* + can1.w:cas9	Naseri et al., 2019
ySCGN01	Gen0.2 + + *pCAROTENE*	This study
ySCGN0.1	*Gen 0.2* + *Pro_*mGAL1*_-NLS-GAL4AD-ATAF1-Pro_*CYC*__1m__*in*__–__*ATAF*__1–4X_-ERG20*, *Pro_*mGAL1*_-NLS-GAL4AD-JUB1*-*Pro_*CYC*__1m__*in*__–__*JUB*__1–2X_- GDH2*, *Pro_*mGAL1*_-*NLS-GAL4AD-ANAC102-*Pro_*CYC*__1m__*in*__–__*ANAC10*__2–2X_ -tHMG1*	This study
ySCGN0.2	*Gen 0.2* + *lpp1a*, *dpp1a, gdh1a*	This study
ySCGN01.2	ySCGN 0.1 + *lpp1a*, *dpp1a, gdh1a*	This study
ySCGN02	ySCGN01.2 + *pCAROTENE*	This study

The SFC data (Figure 5G) demonstrate a ∼4.8-fold higher β-carotene level in the yPPGN022 *P. pastoris* strain than that in the ySCGN01 *S. cerevisiae* strain. As a further control, the episomal pCAROTENE-PGAP plasmid expressing the three genes from the *GAPDH* promoter was transformed into J308 *P. pastoris* strain to generate yPPGN023 strain. The SFC data (Figure 5G) demonstrate that the strain with ATF/BS control modules produced ∼3.4-fold more β-carotene than the strain with *GAPDH* promoters. We observed a relatively huge variation of β-carotene production levels in different replicates in the case of yPPGN23 strain. A common issue often encountered with the episomal expression systems is an unstable product output because of the limited control of copy number, and segregational instability even in selective medium (Futcher and Carbon, 1986). In the case of pCAROTENE-PGAP, the metabolic burden raised from the constitutive expression of the pathway enzymes may cause this effect to be more severe. In contrast, presence of the inducible promoter upstream of plant-derived ATFs allows β-carotene production after adding IPTG and galactose (in the production phase). Therefore, a considerable production of β-carotene was achieved in the case of pCAROTENE, compared to pCAROTENE-PGAP (Figure 5G). Although the production of β-carotene in the ySCGN02 *S. cerevisiae* strain was markedly improved as compared with that in the ySCGN01 wild-type strain (by ∼8.2-fold), it was only ∼1.7-fold higher than the level of β-carotene accumulation in the yPPGN022 strain, confirming the high transcriptional capability of plant-derived ATF/BSs for metabolic engineering applications in *P. pastoris.*

## Discussion

Historically, *S. cerevisiae* is the most often used synthetic biology workhorse for heterologous protein production. Recently, other yeasts such as *K. phaffii*, *Kluyveromyces lactis*, and *Yarrowia lipolytica* have emerged as advantageous cell factories. Among these, *K. phaffii*, commonly known as *P. pastoris*, has gained attention for marketing novel recombinant therapeutics (Chen et al., 2020; Karbalaei et al., 2020; Spice et al., 2020; Zhang et al., 2020). To uncover and navigate the huge potential of *P. pastoris* in bioengineering, we need to establish straightforward strategies to assemble multi-component DNA constructs harboring genes and other essential regulatory elements. This situation decidedly relies on the availability of regulator elements, such as TFs and their *cis*-regulatory elements, which can be combined to establish the synthetic regulatory systems. For example, GoldenPiCS is a modular Golden Gate-derived *P. pastoris* cloning system that allows the creation of complex genetic constructs in a relatively short period of time (Prielhofer et al., 2017). However, genetic tools allowing the orthogonal regulation of cellular activities at diverse levels (DNA replication, transcription, translation, and others) are still not sufficiently established in *P. pastoris* (Kang et al., 2017).

In the past, few native promoters of *P. pastoris* have been used for the controlled expression of heterologous coding sequences in yeast (Waterham et al., 1997; Qin et al., 2011). Although these regulatory sequences allow gene expression over a wide range of activities, control over them is accomplished by endogenous yeast gene regulatory networks ruled by native TFs, which may not be attractive since minimal interference with the host endogenous regulatory network is highly preferred in synthetic biology. Additionally, the most efficient designs ideally incorporate a biomass production phase followed by a target production phase. By establishing a collection of inducible regulators, the expression of regulators, and therefore any potential negative interactions with the host genome, metabolites, and proteins that impose fitness costs, can be postponed until a desired time. We previously established a library of ATF/BSs using several TF families from the higher plant, *Arabidopsis thaliana* (that are absent from non-plant pro- and eukaryotes) to control gene expression in *S. cerevisiae* (Naseri et al., 2017). We hypothesized that such TFs may be principally well-suited for establishing orthologous gene regulatory elements in *P. pastoris*.

Here, we tested 17 different combinations of plant-derived ATFs and their BSs following chromosomal integration and observed a wide spectrum of inducible transcriptional outputs (Figure 2). A low transcriptional output and minimum growth defects observed for plant-derived ATF/BSs in non-induction medium (Figures 2, 4) render them suitable regulators, allowing separation of biomass accumulation from the target molecule production phase. With the exception of EDLLAD-ANAC102-derived ATFs that resulted in a low transcriptional output in *P. pastoris*, we observed a high degree of regulator transferability from *S. cerevisiae* to *P. pastoris*. In other words, the strongest regulators led to the highest mean expression of reporter protein, regardless of the host of choice. To demonstrate a useful application of plant-derived ATF/BSs, we studied β-carotene productivity in *P. pastoris*, where plant-derived ATF/BSs were used to control gene expression. *P. pastoris* was able to produce ∼1.35 mg β-carotene/g (cdw), which is ∼4.8-fold greater than that produced by *S. cerevisiae*. By implementing the CRISPR/Cas9-mediated one-step multigene modification system (Bao et al., 2015) and inducible super-strong plant-derived ATF/BSs (up to 10-fold stronger than the *S. cerevisiae* constitutive *TDH3* promoter) (Naseri et al., 2017), we increased the endogenous supply by *S. cerevisiae* through the redirection of its endogenous metabolic flux toward FPP, a key precursor of a non-native β-carotene product. We proved plant-derived ATFs are a promising tool for fine-tuning the gene expression, either via controlling the heterologous protein production or reviewing the metabolisms of the microbial chassis (Figure 5). This strain can be used for maximized production of other isoprenoid products including squalene (Paradise et al., 2008; Ding et al., 2014). Ito et al. (2020) showed tuneable gene expression in *P. pastoris* using endogenous terminators of *S. cerevisiae* (Ito et al., 2020). Here, we also report the successful transferability of terminators and promoters, such as the *CYC1* minimal promoter, from *S. cerevisiae* to *P. pastoris* (Figure 5A), in addition to plant-derived ATF/BSs.

IPTG is expensive and induction is irreversible. Moreover, galactose (required for IPTG-inducible system) negatively affects yeast cell growth (Figure 5). Therefore, IPTG/galactose responsive *GAL1* promoter is not the best choice for industrial-scale production. The plant-derived ATFs are compatible with other chemically inducible promoters and light-controlled molecular switches that may be more promising alternatives for industrial application (Losi et al., 2018). We tested the functionality of the plant-derived *cis*-regulatory elements only within the frame of the yeast *CYC1* minimal promoter. Another aspect for further refinement is the evaluation of other minimal promoters (Schlabach et al., 2010) from either yeast or plants, which can be established and modified by altering the number of ATF binding sites. This will allow establishment of a vast spectrum of controllable transcriptional regulatory systems, strongly supporting future applications in synthetic biology.

*P. pastoris* is a promising cell factory for the high-level expression of heterologous proteins for industrial and pharmaceutical applications, owing to the growth to high cell densities in mineral medium in batch-fed cultivation (Krause et al., 2010), the possibility of targeting proteins for secretion, and the low amount of secreted endogenous proteins (Barrero et al., 2018). However, engineering microorganisms for industrial-scale production remains a challenge, even if we succeed in establishing cutting-edge synthetic biology tools and assembly methods. In other words, the use of multiple circuits to build functional complex systems is a demanding task, and a large number of diverse synthetic constructs need to be created. This situation strongly demands parallel and smart methods, referred to as combinatorial optimization methods, allowing the simultaneous assembly of large numbers (up to thousands or even millions) of variants (Naseri and Koffas, 2020). The plant-derived ATF/BSs could be employed in the frame of combinatorial optimization methods, such as our COMPASS (Naseri et al., 2019; Naseri and Mueller-Roeber, 2020), for pathway optimization in *P. pastoris*. Our ATF/*cis*-regulatory library therefore represents an excellent starting point for further improvements to orthogonal regulatory systems in yeast.

## Data Availability Statement

The original contributions presented in the study are included in the article/Supplementary Material, further inquiries can be directed to the corresponding author/s.

## Author Contributions

GN conceived the project, designed, performed the experiments, analyzed the data, and wrote the manuscript. CA designed SFC experiment. KP, HH, and CA performed and analyzed SFC experiment. All authors read and approved the manuscript.

## Conflict of Interest

The authors declare that the research was conducted in the absence of any commercial or financial relationships that could be construed as a potential conflict of interest.

## Publisher’s Note

All claims expressed in this article are solely those of the authors and do not necessarily represent those of their affiliated organizations, or those of the publisher, the editors and the reviewers. Any product that may be evaluated in this article, or claim that may be made by its manufacturer, is not guaranteed or endorsed by the publisher.
