# Supplementary material for: Artificial Transcription Factors for Tuneable Gene Expression in Pichia pastoris
Source: Front Bioeng Biotechnol. 2021 Aug 9;9:676900. doi: 10.3389/fbioe.2021.676900 (PMC8381338; doi:10.3389/fbioe.2021.676900)
Supplement: Supplementary file 12 [file Image_1.pdf]

Supplementary Figures

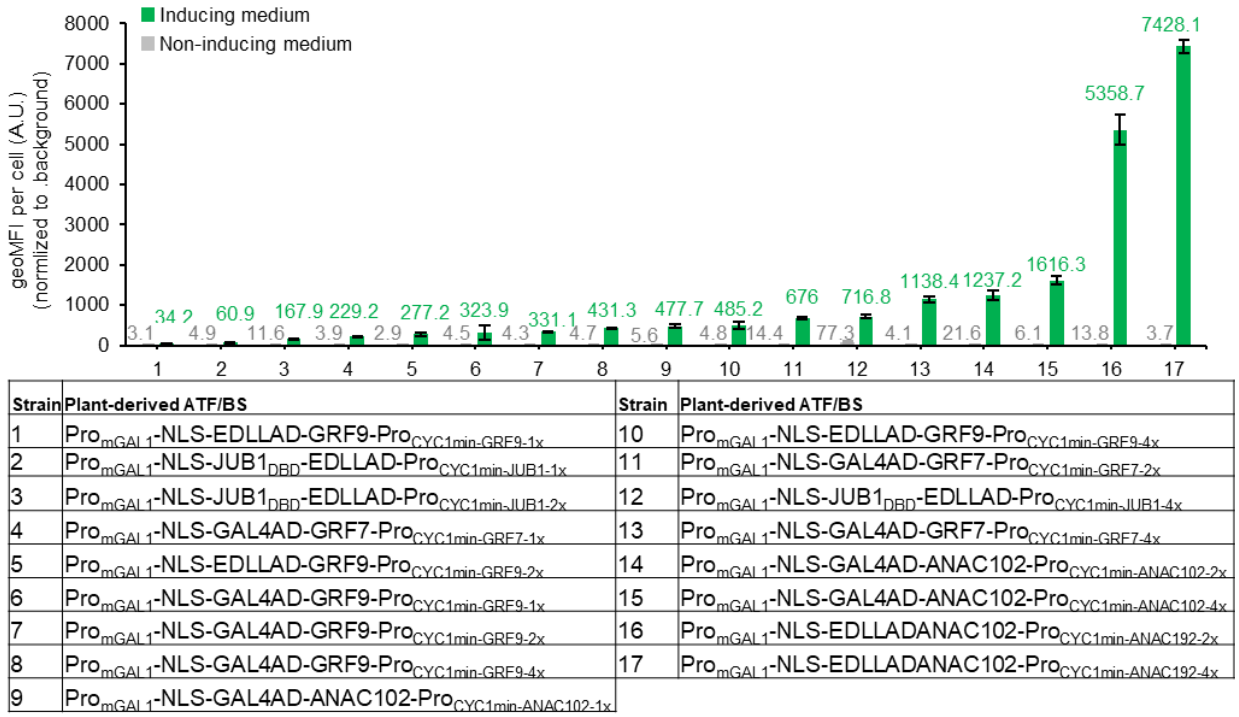

Strain	Plant-derived ATF/BS	Strain	Plant-derived ATF/BS
1	Pro$_{mGAL1}$-NLS-EDLLAD-GRF9-Pro$_{CYC1min-GRF9-1x}$	10	Pro$_{mGAL1}$-NLS-EDLLAD-GRF9-Pro$_{CYC1min-GRF9-4x}$
2	Pro$_{mGAL1}$-NLS-JUB1$_{DBD}$-EDLLAD-Pro$_{CYC1min-JUB1-1x}$	11	Pro$_{mGAL1}$-NLS-GAL4AD-GRF7-Pro$_{CYC1min-GRF7-2x}$
3	Pro$_{mGAL1}$-NLS-JUB1$_{DBD}$-EDLLAD-Pro$_{CYC1min-JUB1-2x}$	12	Pro$_{mGAL1}$-NLS-JUB1$_{DBD}$-EDLLAD-Pro$_{CYC1min-JUB1-4x}$
4	Pro$_{mGAL1}$-NLS-GAL4AD-GRF7-Pro$_{CYC1min-GRF7-1x}$	13	Pro$_{mGAL1}$-NLS-GAL4AD-GRF7-Pro$_{CYC1min-GRF7-4x}$
5	Pro$_{mGAL1}$-NLS-EDLLAD-GRF9-Pro$_{CYC1min-GRF9-2x}$	14	Pro$_{mGAL1}$-NLS-GAL4AD-ANAC102-Pro$_{CYC1min-ANAC102-2x}$
6	Pro$_{mGAL1}$-NLS-GAL4AD-GRF9-Pro$_{CYC1min-GRF9-1x}$	15	Pro$_{mGAL1}$-NLS-GAL4AD-ANAC102-Pro$_{CYC1min-ANAC102-4x}$
7	Pro$_{mGAL1}$-NLS-GAL4AD-GRF9-Pro$_{CYC1min-GRF9-2x}$	16	Pro$_{mGAL1}$-NLS-EDLLADANAC102-Pro$_{CYC1min-ANAC192-2x}$
8	Pro$_{mGAL1}$-NLS-GAL4AD-GRF9-Pro$_{CYC1min-GRF9-4x}$	17	Pro$_{mGAL1}$-NLS-EDLLADANAC102-Pro$_{CYC1min-ANAC192-4x}$
9	Pro$_{mGAL1}$-NLS-GAL4AD-ANAC102-Pro$_{CYC1min-ANAC102-1x}$		

Supplementary Figure 1.

The genome-integrated, plant-based ATFs in *S. cerevisiae*.

A total of 17 different plant-derived ATFs and promoter pair combinations, providing weak (1–7; 0–400 AU), medium (8–13; 400–1,200 AU), and strong (14–17; 1,200–8,000 AU) transcriptional outputs (green) and low basal expression (grey), were selected from the previously characterised library of plant-derived ATF/BS integrated into the *ura3-52* locus. geoMFI, geometric mean fluorescence intensity; AU, arbitrary units, determined using EGFP as a reporter. ANAC102, A NAC domain transcription factor; GRF7, Growth-Regulating Factor 7; GRF9, Growth-Regulating Factor 9; JUB1, JUNGBRUNNEN1; NLS, nuclear localization signal; Pro$_{CYC1mini}$, *CYC1* minimal promoter; Pro$_{mGAL1}$, modified *GAL1* promoter. ´1X´, ´2X´, and ´4X´ indicate the number of bindings sites implemented for the ATFs within the *CYC1* minimal promoters. Data are shown in **Data S5**.
